# *Pseudomonas fluorescens* SBW25 produces furanomycin, a non-proteinogenic amino acid with selective antimicrobial properties

**DOI:** 10.1186/1471-2180-13-111

**Published:** 2013-05-20

**Authors:** Kristin Trippe, Kerry McPhail, Donald Armstrong, Mark Azevedo, Gary Banowetz

**Affiliations:** 1 USDA-ARS National Forage Seed Production Research Center, Corvallis, OR, 97331, USA; 2 College of Pharmacy, Oregon State University, Corvallis, OR, 97331, USA; 3 Department of Botany and Plant Pathology, Oregon State University, Corvallis, OR, 97331, USA

**Keywords:** *Pseudomonas fluorescens* SBW25, *Pseudomonas fluorescens* WH6, Secondary metabolites, Non-proteinogenic amino acids, Antimicrobial activity, 4-formylaminooxyvinylglycine, Furanomycin

## Abstract

**Background:**

*Pseudomonas fluorescens* SBW25 has been extensively studied because of its plant growth promoting properties and potential as a biocontrol agent. The genome of SBW25 has been sequenced, and among sequenced strains of pseudomonads, SBW25 appears to be most closely related to *P. fluorescens* WH6. In the authors’ laboratories, WH6 was previously shown to produce and secrete 4-formylaminooxyvinylglycine (FVG), a non-proteinogenic amino acid with selective herbicidal and antimicrobial activity. Although SBW25 does not have the genetic capacity to produce FVG, we were interested in determining whether this pseudomonad might produce some other type of non-proteinogenic amino acid.

**Results:**

*P. fluorescens* SBW25 was found to produce and secrete a ninhydrin-reactive compound with selective antimicrobial properties. This compound was purified from SBW25 culture filtrate and identified as the non-proteinogenic amino acid L-furanomycin [2S,2′R,5′S)-2-amino-2-(5′methyl-2′,5′-dihydrofuran-2′-yl)acetic acid].

**Conclusions:**

The identification of furanomycin as a secondary metabolite of SBW25 is the first report of the production of furanomycin by a pseudomonad. This compound was known previously only as a natural product produced by a strain of *Streptomyces*. This report adds furanomycin to the small list of non-proteinogenic amino acids that have been identified as secondary products of pseudomonads. This study also extends the list of bacteria that are inhibited by furanomycin to include several plant pathogenic bacteria.

## Background

*Pseudomonas fluorescens* is a γ –proteobacterium that is found throughout terrestrial ecosystems but is most commonly isolated from the surface of plant roots and leaves. Strains of *P. fluorescens* are physiologically and ecologically diverse, representing at least five biovars [1]. The extreme heterogeneity among *P. fluorescens* isolates has led scientists to propose that strains of *P. fluorescens* form a complex of species [1-3]. Recent analyses that compare the genomes of several *P. fluorescens* strains support that hypothesis [4] and demonstrate that strains of *P. fluorescens* arose from at least three separate lineages [5].

The large genomes of *P. fluorescens* provide an extensive biochemical repertoire that enables some strains to produce and secrete bioactive molecules that mediate microbe-microbe, plant-microbe, and insect-microbe interactions [6]. These secondary metabolites include antimicrobial compounds like phenazines, polyketides, cyclic lipopeptides, pyrrolnitrin, hydrogen cyanide, and others [6,7]. Because these compounds may play a critical role in both microbial and plant ecology, there is continuing interest in characterizing secondary metabolites produced by isolates of *P. fluorescens.*

*P. fluorescens* WH6, a strain originally isolated from the rhizosphere of wheat [8,9], has been shown in our laboratories to produce and secrete a low molecular weight compound that has selective herbicidal and antimicrobial properties [10,11]. This compound, which we termed a Germination-Arrest Factor (GAF), selectively and irreversibly arrests the germination of a large number of graminaceous species, including a number of invasive grassy weeds [10]. We identified GAF as the non-proteinogenic amino acid 4-formylaminooxyvinylglycine (FVG, L-2-amino-4-formylaminooxy-*trans-*3-butenoic acid) [12]. FVG was subsequently shown to also have selective antimicrobial activity against a few strains of bacteria (13), including *Erwinia amylovora*, the bacterial plant pathogen that is the causal agent of the disease of orchard crops known as fireblight.

The genome of *P. fluorescens* WH6 has been sequenced [13] and compared to other sequenced strains of *P. fluorescens*[5,13]. Among sequenced strains of pseudomonads, these comparative genomic and phylogenetic analyses indicated that WH6 was most closely related to SBW25. These two strains appear to represent a distinct clade within the lineage that includes *P. fluorescens* A506 and BG33R [5]. These analyses have shown that 69% of *P. fluorescens* WH6 genes have an orthologous sequence in SBW25, and they share extensive long-range synteny [13]. Nonetheless, in spite of the overall similarity of the SBW25 genome to that of WH6, SBW25 lacks a gene cluster we have shown to be essential to the biosynthesis of FVG [14].

*P. fluorescens* SBW25 was first isolated from the leaf surface of a sugar beet plant [15]. Since then it has been used as a model organism for evolutionary and plant colonization studies [16-20]. SBW25 has also been extensively studied for its plant growth-promoting properties and its ability to protect peas from seedling damping-off caused by the oomycete *Pythium ultimatum*[21]. The secondary metabolites known to be produced by SBW25 include pyoverdine siderophores [22] and a viscosin-like cyclic lipopeptide [23]. The latter compound exhibits zoosporicidal activity towards a different oomycete, *Phytophthora infestans*, but its primary role appears to be in biofilm formation and facilitating the surface motility of SBW25 [23].

Although the *P. fluorescens* SBW25 genome does not contain the gene cluster we have found to be essential for FVG production, the overall similarity of the WH6 and SBW25 genomes attracted our interest in the latter strain and in the possibility that SBW25 might also produce some type of non-proteinogenic amino acid. In the present study, we report that *P. fluorescens* SBW25 produces and secretes a ninhydrin-reactive compound that selectively inhibits the growth of several bacterial plant pathogens. This compound was purified from *P. fluorescens* SBW25 culture filtrates and identified as the amino acid L-furanomycin. To our knowledge, this is only the second report of furanomycin production by a microbe and the first report of furanomycin production by a pseudomonad.

## Results

### Presence of ninhydrin-reactive compounds in *P. fluorescens* SBW25 culture filtrate

**Figure 1:**
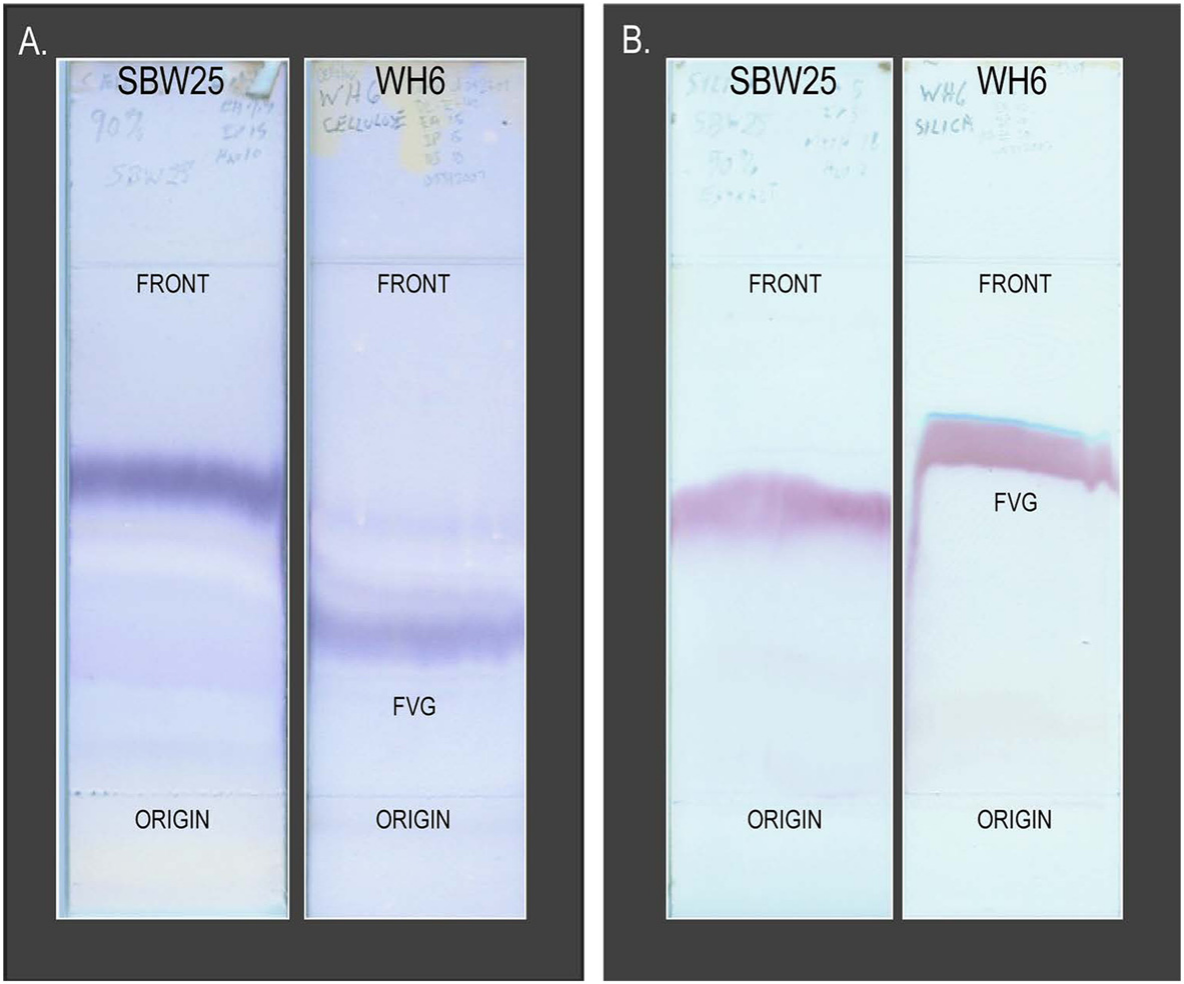
**Thin-layer chromatograms of 90% ethanol extracts of dried culture filtrates from *P. fluorescens *SBW25 and WH6. A**. Ninhydrin-stained cellulose TLC chromatograms. **B**. Ninhydrin-stained GHL-silica TLC chromatograms. Sample preparation and application and the solvent systems used to develop the chromatograms are described in the Methods section. The ninhydrin band corresponding to FVG is indicated on the WH6 chromatogram.

As a preliminary test for the production of non-proteinogenic amino acids by *P. fluorescens* SBW25, and to compare SBW25 culture filtrates with filtrate from WH6, dried culture filtrates of SBW25 and WH6 were extracted with 90% ethanol. Aliquots of the concentrated extracts were fractionated by thin-layer chromatography (TLC) on cellulose and silica plates. The resulting chromatograms were then stained with ninhydrin (Figure 1). The extract of SBW25 culture filtrate yielded a single, strongly-staining, ninhydrin-reactive band on both cellulose and silica TLC plates. On cellulose TLC plates, this band exhibited greater mobility than the FVG band visible in the chromatogram of the extract from WH6. On silica TLC plates, the SBW25 band had a slightly lower R_f_ than FVG from WH6.

### Biological activity of *P. fluorescens* SBW25 culture filtrate

The antimicrobial properties of *P. fluorescens* SBW25 culture filtrate were tested against a panel of bacteria that included multiple races, pathovars, and strains of eleven bacterial species (Table 1). Of the nineteen bacterial strains tested in our standard agar diffusion assay, six were sensitive to the filtrate as evidenced by a large zone of clearing about the central well containing the SBW25 culture filtrate. These six strains are listed in Table 2 and included five plant pathogens. Typical results of the agar diffusion assay are illustrated in Additional file 1, which shows results with five of the sensitive strains and one of the insensitive strains tested. Of the strains inhibited by SBW25 culture filtrate, *P. syringae* pv. *tomato* DC3000 was the most sensitive. However, because *P. syringae* pv*. tomato* DC3000 harbors its own antibacterial properties [24], we chose *Dickeya dadantii* 1447, a pathovar that causes bacterial soft rot of corn, to use in following antibiotic activity in subsequent purification work. The bacterial plant pathogens inhibited by SBW25 culture filtrate included *Erwinia amylovora,* which is also selectively inhibited by culture filtrate from *P. fluorescens* WH6 [25]. However, unlike WH6, SBW25 culture filtrate did not inhibit the germination of *Poa annua* in our standard germination arrest assay [10].

**Table 1:** **Bacterial strains tested for sensitivity to *P. fluorescens *SBW25 filtrate**

**Test species**	**Strain**	**Source of strain**
*Agrobacterium tumefaciens*	C58bv1	2
*Bacillus megaterium*	K2	1
*Dickeya dadantii*	X179	3
	1447	3
*Erwinia amylovora*	153	1
*Escherichia coli*	HB101	5
	DH5α	5
*Pantoea agglomerans*	EH252	1
*Pectobacterium carotovora*	cc101	1
*Pseudomonas fluorescens*	A506	1
	D7	6
	WH6	7
*Pseudomonas marginalis*	PM-7	2
*Pseudomonas syringae*	*glycinea* race 0, 4	4
	*phaseolicola* 1448A	4
	*maculicola* M4	4
	*tomato* DC3000	4
*Stenotrophomonas maltophilia*	RM145	2

^1^Dr. Joyce Loper (USDA-ARS, Corvallis, OR, USA).

^2^Marilyn Miller (Plant Clinic, Dept. of Botany & Plant Pathology, Oregon State University, Corvallis, OR, USA).

^3^ Dr. Kenneth Johnson (Dept. of Botany & Plant Pathology, Oregon State University, Corvallis, OR, USA).

^4^Dr. Jeff Chang (Department of Botany & Plant Pathology, Oregon State University, Corvallis, OR, USA).

^5^commercial source.

^6^Dr. Ann Kennedy (USDA-ARS, Pullman, WA, USA).

^7^Dr. Gary Banowetz (USDA-ARS, Corvallis, OR, USA).

**Table 2:** **Bacteria that are sensitive to *P. fluorescens *SBW25 culture filtrate**

**Test species**	**Strain**	**Zone size (cm**^**2**^**)**
*Bacillus megaterium*	K2	15.3 ± 0.22
*Dickeya dadantii*	X179	6.7 ± 0.29
	1447	10.1 ± 0.57
*Erwinia amylovora*	153	13.5 ± 0.34
*Pseudomonas syringae*	*maculicola* M4	12.2 ± 1.45
	*tomato* DC3000	31.0 ± 0.97

The sizes of the zones of clearing produced in the lawns of bacteria surrounding the central well containing the filtrate are indicated.

### Association of the antimicrobial activity of SBW25 culture filtrate with a ninhydrin-reactive compound

**Figure 2:**
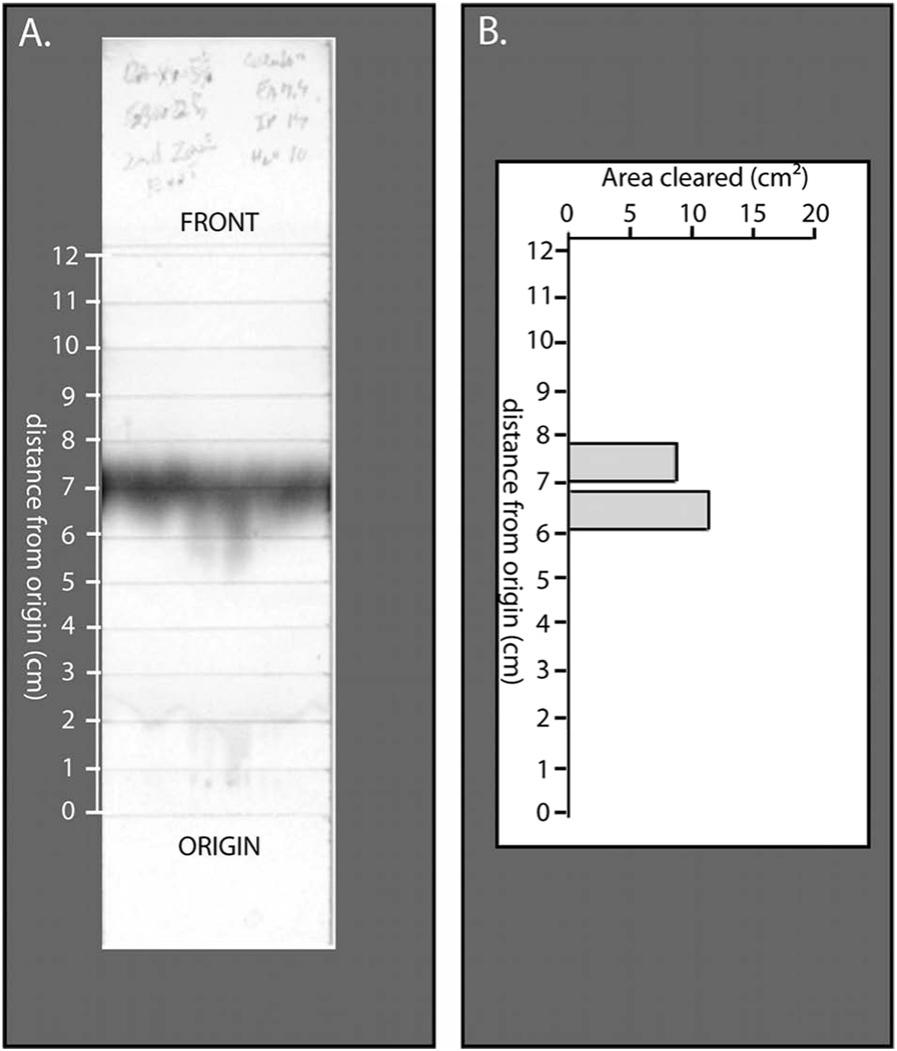
**The distribution of antimicrobial activity and ninhydrin-banding after TLC fractionation of an 85% ethanol extract of dried culture filtrate from *P. fluorescens *SBW25.** The 85% ethanol extract was prepared and applied to cellulose TLC plates as described in Methods. One of the developed plates was sprayed with ninhydrin (Figure 2A) and the replicate plate was divided into zones, as indicated, for removal and extraction of the cellulose. The aqueous extracts of the cellulose from each zone were assayed for antimicrobial activity according to the standard assay described in the Methods section. The resulting antimicrobial activity against *Dickeya dadantii* (Figure 2B) was measured after 48 h. Zones without bars did not result in a cleared zone when assayed with either *D. dadantii* or *P. syringae* pv. *maculicola* M4.

The possibility that the antimicrobial activity of SBW25 culture filtrates was associated with the ninhydrin-reactive component of the filtrate was examined in additional fractionation studies. Preliminary experiments determined that most of the ninhydrin-reactive compound from SBW25 culture filtrate was extracted from the dried culture filtrate solids by extraction with 85% ethanol. To determine if the antimicrobial activity of *P. fluorescens* SBW25 culture filtrate could be attributed to the ninhyrin-reactive component of the filtrate, aliquots of the 85% ethanol extract were fractionated on replicate cellulose TLC plates. One of the chromatograms was stained with ninhydrin, and the remaining cellulose plate was divided into twelve 1-cm zones that were then extracted with water. The resulting extracts were tested for antimicrobial activity in our standard assay. All of the antimicrobial activity towards *D. dadantii* 1447 was coincident with the position of the ninhydrin-band on the replicate plate (Figure 2). Similar results were obtained with *P. syringae* pv. *maculicola* M4.

### Purification of the ninhydrin-reactive component of SBW25 culture filtrate

**Figure 3:**
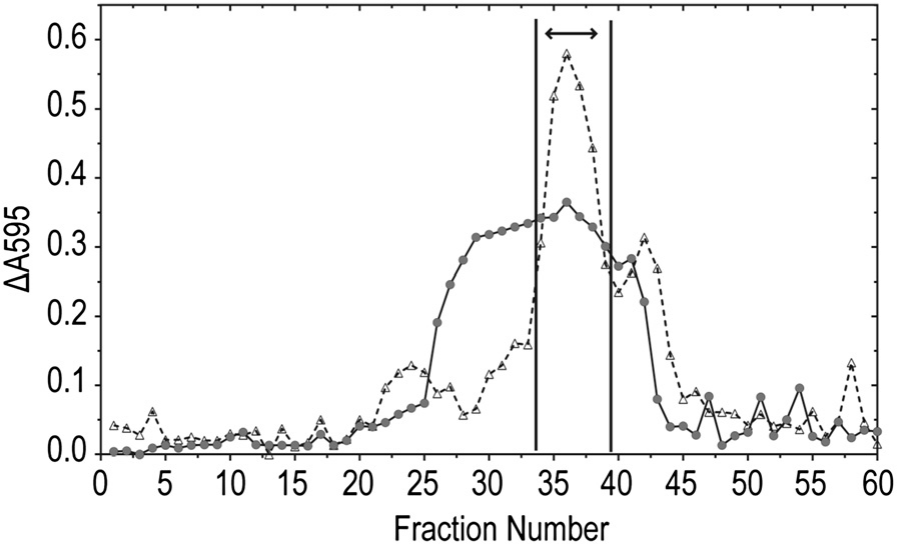
**Initial Sephadex G-15 column fractionation of an 85% ethanol extract of dried culture filtrate from *Pseudomonas fluorescens *SBW25.** The solids from 840 mL of dried SBW25 culture filtrate were extracted with 85% ethanol as described in the Methods section. A portion of the extract equivalent to 800 mL of original culture filtrate was taken to dryness *in vacuo* and dissolved in 6 mL of deionized water for application to a Sephadex G-15 column equilibrated in the same solvent. The column was eluted with deionized water. Fractions (6 mL each) were collected and analyzed for reaction with the Fe- and Cu-CAS reagents as described in the Methods section. The fractions corresponding to the largest Cu-binding peak were pooled (as indicated by the double arrow) for concentration and further purification by preparative TLC fractionation.

Purification of the ninhydrin-reactive compound from *P. fluorescens* SBW25 culture filtrate was undertaken by a modification of the strategy used by McPhail *et al.*[12] to purify FVG. SBW25 culture filtrate (840 mL) was taken to dryness *in vacuo,* and the dried solids were extracted with 85% ethanol. Following evaporation of the 85% ethanol extract, the solids were dissolved in a small volume of water and applied to a Sephadex G-15 column equilibrated with deionized water for fractionation by ion exclusion chromatography. The elution profile of this column (Figure 3) was monitored by assaying aliquots of each column fraction with ChromeAzurol reagents according to the protocol previously developed by McPhail *et al.*[12]. The profile exhibited a distinct peak of Cu-binding activity (expected to correspond to compounds containing amino groups) followed by a smaller peak, both of which overlapped an extended peak of Fe-binding activity (reflecting the elution of contaminating phosphate from the culture medium). The fractions corresponding to the larger peak of Cu-binding activity were pooled, taken to dryness *in vacuo*, and the recovered solids dissolved in 76% ethanol for preparative TLC fractionation. Following preparative TLC, the area on the TLC plate corresponding to the position of the ninhydrin-reactive compound was scraped from each plate and extracted with deionized water. The combined aqueous extracts were dried *in vacuo* and dissolved in a small volume of deionized water for rechromatography on a Sephadex G-15 column.

**Figure 4:**
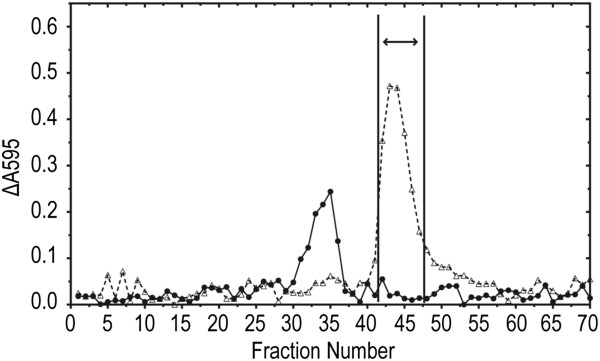
**Final Sephadex G-15 column purification of the partially purified ninhydrin-reactive compound recovered from preparative TLC chromatograms.** A sterile aqueous solution containing the partially purified SBW25 ninhydrin-reactive compound was prepared by extraction of the appropriate zone of preparative TLC chromatograms as described in the Methods section. This solution was taken to dryness *in vacuo*, and the recovered solids were dissolved in 5 mL of deionized water for application to the Sephadex G-15 column. The column was eluted with deionized water. Fractions (5 mL each) were collected and analyzed for reaction with the Fe- and Cu-ChromeAzurol S reagents. The fractions corresponding to the Cu-binding peak were pooled (as indicated by the double arrow) and concentrated for structural identification.

The elution profile for Sephadex G-15 column fractionation of the material recovered from preparative TLC purification exhibited a Cu-binding peak that was clearly separated from a smaller Fe-binding peak, indicating that the ninhydrin-reactive compound was separated from the contaminating phosphate (Figure 4). The fractions from the Cu-binding peak were pooled as indicated, and an aliquot of this pooled material was tested for antimicrobial activity in agar diffusion assays*.* The tested aliquot strongly inhibited the growth of *D. dadantii 1447.* The pooled fraction was then taken to dryness and re-dissolved in 76% ethanol. TLC analysis of an aliquot of the 76% solution gave a single ninhydrin-staining band at the expected R_f_, and no UV-absorbing or fluorescent compounds were detected. The remainder of the 76% ethanol solution of the purified compound, corresponding to *ca.* 600 mL of original culture filtrate, was concentrated *in vacuo* and yielded 3.7 mg of a white amorphous solid, of which 3.6 mg was dissolved in 500 μL of deuterated water (D_2_O) for nuclear magnetic resonance (NMR) analysis. The remaining 0.1 mg was submitted for high-resolution electrospray mass spectrometry (HRESIMS) to determine molecular composition.

### Identification of the purified ninhydrin-reactive compound

**Figure 5:**
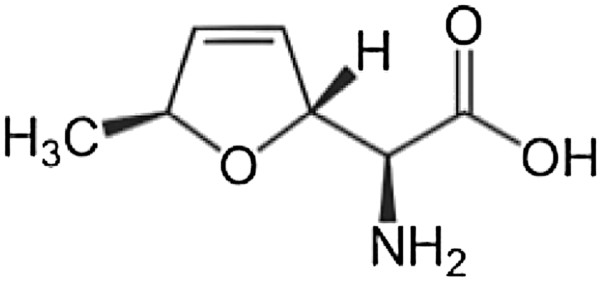
Molecular structure of L-furanomycin.

HRESIMS data for the purified compound provided a molecular ion [M+H]^+^ at *m/z* 158.0812. Examining the microbial natural products database Antibase 2011, the Natural Compound Identifier (Wiley-VCH) reported 11 nitrogen-containing compounds from a search of the mass range 157.0 to 157.5 Da. Six of these were alpha amino acids. Inspection of the ^1^H NMR spectrum (Additional file 2) for the purified compound revealed an upfield methyl doublet (δ_H_ 1.14, 3H), and five deshielded multiplets consistent with five heteroatom-substituted or olefinic methines (δ_H_ 6.07, 5.74, 5.34, 5.00 and 3.75, each 1H). These six signals were correlated in a single spin system as judged from the COSY spectrum. Two additional complex multiplets appearing mid-field in the ^1^H NMR spectrum did not integrate to relative integer values, and showed no COSY correlations to the established spin system. In combination with two additional mid-field ^13^C resonances in the ^13^C NMR spectrum (Additional file 3) these ^1^H signals could be attributed to contaminating glycerol and discounted from further consideration. The ^13^C NMR spectrum also showed a quaternary ^13^C signal (δ_C_ 172.3), as well as *Heteronuclear Single Quantum Coherence*-correlated resonances for five methines and one methyl carbon in the purified compound. The methine ^13^C chemical shifts represented two olefinic carbons (δ_C_ 136.3 and 124.3), two oxygenated carbons (δ_C_ 84.31 and 84.24), and an amine-substituted carbon (δ_C_ 57.5). In combination with the HREIMS data, these NMR data support a molecular formula of C_7_H_11_NO_3_ and the molecular structure of the alpha amino acid furanomycin (also known as threomycin) [26]. As anticipated, the NMR data for the purified compound matched closely with those reported for L-furanomycin [27] and differed significantly from those for four reported synthetic diastereomers [28,29]. Therefore, the 2*S*, 2′*R*, 5′*S* configuration of L-furanomycin could be assigned, although the presence of the small amount of contaminating glycerol prevented an accurate optical rotation value from being assigned. The molecular structure of L-furanomycin is shown in Figure 5.

### Reversal of the antimicrobial activity of SBW25 culture filtrate with selected amino acids

**Figure 6:**
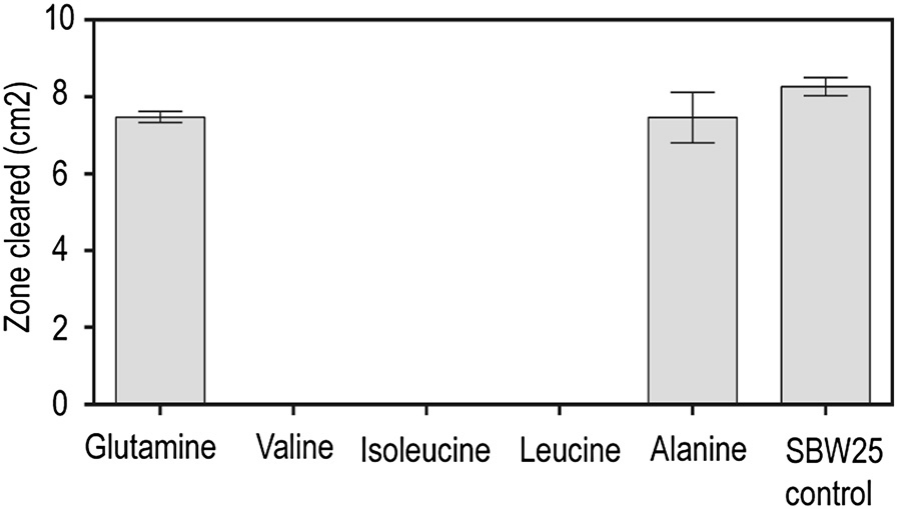
**The effect of selected amino acids on the antimicrobial activity of furanomycin.** The indicated amino acids were added to aliquots of *P. fluorescens* SBW25 culture filtrate to give a final amino acid concentration of 10 mM. The resulting solutions were filter sterilized and tested for antimicrobial activity against *D. dadantii* in our agar diffusion assay as described in the Methods section. The areas of the cleared zones in the bacterial lawns surrounding the central well containing the test solutions are the averages of three replicates. The error bars represent Standard Error of the Mean values.

The ability of furanomycin to inhibit the growth of various bacteria was reported to be reversed by the amino acids leucine, isoleucine, or valine [26]. To determine if the mode of action of furanomycin in inhibiting plant pathogenic bacteria is similar to the mode of action previously described, we added these individual amino acids to SBW25 culture filtrates (10 mM final concentration) and assayed their ability to inhibit the growth of *D. dadantii* 1447. Glutamine, alanine, and serine, which we had found previously to reverse the effects of FVG in inhibiting the growth of *Erwinia amylovora*, were also tested in this manner. *D. dadantii* 1447 was not sensitive to SBW25 culture filtrate containing isoleucine, leucine, or valine (Figure 6, Additional file 4). However, *D. dadantii* remained sensitive to SBW25 culture filtrate supplemented with glutamine or alanine and to the unmodified filtrate control (Figure 6, Additional file 4). These results indicate that the capacity of *P. fluorescens* SBW25 culture filtrate to inhibit the growth of *D. dadantii* 1447 was reversed in the presence of leucine, isoleucine, and valine, but not glutamine or alanine. The ability of serine to block antimicrobial activity in these tests was less clear. When serine was added to the culture filtrate, smaller zones of reduced lawn density were observed. However, because these zones were difficult to measure, the data were not included in our statistical analyses.

## Discussion

The identification of furanomycin in *P. fluorescens* SBW25 culture filtrate is the first report of this compound occurring as a natural product of a pseudomonad. Previously, *Streptomyces threomyceticus* ATCC 15795 was the only microbe known to produce this antibiotic [26]. The biosynthesis of furanomycin in *S. threomyceticus* was investigated by Parry and co-workers [30,31], who obtained evidence from feeding studies that the synthesis proceeded via a polyketide pathway that originated from propionate and acetate. Subsequent chemical mutagenic studies suggested that the pathway involved at least six intermediate steps after assembly of the polyketide chain [32]. However, the specific genes affected by these mutations were not identified. The pathway in SBW25 has yet to be investigated.

The identification of furanomycin as a secondary metabolite of *P. fluorescens* SBW25 adds to a small list of non-proteinogenic amino acids that are known to be produced and secreted by pseudomonads. In addition to furanomycin, this list includes FVG, produced by WH6 [12], rhizobitoxine (4-(2-amino-3-hydroxypropoxy) vinylglycine), produced by *P. andropogonis*[33], methoxyvinylglycine (MVG, L-2-amino-4-methoxy-*trans*-3-butenoic acid), produced by *P. aeruginosa (*ATCC-7700) [34,35], and 3-methylarginine, produced by *P. syringae* pv*. syringae*[36]. We have observed that a number of other strains of pseudomonads produce and secrete ninhydrin-reactive compounds that may represent non-proteinogenic amino acids, but these compounds have yet to be identified.

The non-proteinogenic amino acids identified as secondary metabolites of pseudomonads all display some type of selective antimicrobial properties in *in vitro* tests. For example, FVG and MVG selectively inhibit the growth of *Erwinia amylovora*, the causal agent of fireblight, an important disease of roseaceous orchard crops [25,37]. MVG also inhibits growth of *Acanthamoeba castellanii*[38] and *Bacillus sp.* 1283B [35]. Likewise, 3-methylarginine suppresses the growth of *P. syringae* pv*. glycinia,* the causal agent of bacterial leaf blight [36]. Furanomycin has been shown previously to strongly inhibit T-even coliphage, as well as the growth of several microorganisms (*Shigella paradysenteriae, Salmonella paratyphi* A*,* and *Bacillus subtilis*) [26]. Our study expands the known range of bacteria that are susceptible to furanomycin to include several plant pathogens, including *D. dadantii, P. syringae,* and *E. amylovora,* as well as the nonpathogenic strain *Bacillus megaterium*. The specificity of these effects is of particular interest in relation to the potential utility of these organisms for the biocontrol of plant pathogens.

The production of furanomycin by SBW25 appears to account for the selective antibacterial activities of the culture filtrates from this organism grown under our culture conditions. The reversal of these effects in the presence of isoleucine is consistent with previous reports that this antibiotic functions as an isoleucine analog [26] and is recognized by the isoleucyl-tRNA synthetase from *Escherichia coli*, where it is charged to isoleucine-tRNA and interferes with protein synthesis in that organism [39]. It is less obvious why valine and leucine also interfere with the antibiotic activity of furanomycin, but it is possible that furanomycin interferes with the biosynthesis of branched-chain amino acids, and the presence of an exogenous source of isoleucine, leucine, or valine reverses or compensates for this interference. Whether the numerous pseudomonads and bacterial strains that are insensitive to furanomycin possess isoleucyl-tRNA synthetase activities of more stringent specificity or resist the effects of this antibiotic by other mechanisms remains to be determined.

In addition to their antimicrobial effects, some of the amino acid analogs produced by pseudomonads elicit a response in higher plants. As noted previously, FVG, produced by *P. fluorescens* WH6, inhibits germination of a large number of graminaceous species [10]. Rhizobitoxine can either act as a phytotoxin (when produced by the plant pathogen *Burkholderia andropogonis*), or it can facilitate nodulation in host legumes (when produced by the symbiotic nitrogen-fixing bacterium *Bradyrhizobium elkanii*) [40]. It is not yet known if furanomycin mediates any of the plant growth promoting properties of SBW25 or if it is involved in any other type of plant-microbe interaction.

The biological role that non-proteinogenic amino acids play in pseudomonad physiology and ecology in natural environments has yet to be defined. Phenazine antibiotics have been reported to contribute to the ecological competence of pseudomonads in soil habitats [41], but it is uncertain whether the antimicrobial activities of furanomycin and other amino acid analogs, or the observed effects of some of these compounds on plant growth, are important in natural settings. This class of pseudomonad secondary metabolites has received limited attention to date, and further investigations will be needed to determine their function and importance.

## Conclusions

The results of this study demonstrate that the secondary metabolites produced by *P. fluorescens* SBW25 includes the non-proteinogenic amino acid known as L-furanomycin. This compound is shown here to inhibit the growth of several bacterial strains, including a number of plant-pathogenic microbes. Previously, furanomycin was only known to be produced by a single strain of *S. threomyceticus.* The antimicrobial activity of furanomycin observed here was reversed in the presence of exogenous leucine, isoleucine, and valine, which is consistent with the previously reported ability of this compound to act as an isoleucine antagonist. This study adds furanomycin to the small group of non-proteinogenic amino acids that are known to be produced by pseudomonads, suggesting that these compounds may have a more ubiquitous presence and a more universal role in pseudomonad ecology than has been previously recognized.

## Methods

### Chemicals and chromatographic materials

All aqueous ethanol solutions were prepared from 95% v/v ethanol that had been redistilled prior to use. All other solvents were purchased as spectrophotometric grade reagents. Chrome Azurol S (CAS), 2-(N-morpholino) ethanesulfonic acid (MES), ninhydrin, and Sephadex G-15 (medium grade) were purchased from Sigma-Aldrich (St. Louis, MO). All TLC plates were purchased from Analtech, Inc. (Newark, DE).

### Biological materials

Sources of the bacterial species and strains used in this study are listed in Table 1. All strains were maintained at −80°C in Luria-Bertani liquid medium (LB medium) [42] containing a final concentration of 15% (v/v) glycerol.

Annual bluegrass seeds (*Poa annua* L.) were obtained from 1996 mid-Willamette Valley grass seed screenings and were provided by International Seeds, Halsey, OR, and by C and R Farm, Tangent, OR. Prior to use, the seeds were cleaned to remove straw and seeds of other species.

### Culture filtrate production

*Pseudomonas fluorescens* cells were inoculated into the modified *Pseudomonas* Minimal Salts Medium (PMS medium) described by Banowetz *et al*. [10], and cultured and harvested as described in the same reference. To prepare culture filtrates, the 7-day *P. fluorescens* cultures were centrifuged (3000 × g, 15 min), and the supernatant was passed through a bacteriological filter (Millipore GP Express Steritop, 0.22 μM pore size, Millipore, Billerica, MA). The resulting sterile culture filtrate was stored at 4°C prior to use.

### Agar diffusion assays for antimicrobial activity

To test the antimicrobial activity of *P. fluorescens* SBW25 filtrate, bacterial strains were grown overnight in LB medium (6 mL) at 28°C (except for *Escherichia coli*, which was grown at 37°C) with shaking (225 rpm). The following morning, the stationary phase bacterial suspensions were adjusted with sterile water to an optical density of 0.2 at 600 nm (or 0.8 in the case of *E. coli*) as measured with a Superspec 3000 (Biorad Inc., Hercules, CA). A 300-μL aliquot of the diluted culture was spread onto the surface of a 925 Minimal Medium plate (100 × 15 mm, containing 25 mL of medium). The 925 Minimal Medium [43] was prepared with the modifications described by Halgren *et al.*[25]. After spreading the bacterial lawn, central wells were punched in the agar with a No. 9 cork borer, and a 300-μL aliquot of SBW25 culture filtrate was dispensed into the well. The plates were incubated for 48 h at 28°C, examined, and scored. Zones of inhibition in the area adjacent to the well were quantified with Able Image Analyzer® software (MU Labs, Ljublijana, Slovenia). Three replicate plates were prepared for each bacterial strain tested, and the experiment was repeated for any strain that appeared sensitive to the SBW25 filtrate.

### Germination arrest assays

The ability of SBW25 culture filtrate to inhibit the germination of *Poa annua* seeds was tested according to the protocol described by Banowetz *et al.*[10].

### Ethanol extraction of culture filtrate

Measured volumes of *P. fluorescens* culture filtrate were taken to dryness *in vacuo* at a temperature ≤ 45°C. After evaporation, the dry solids were extracted three times (5 min per extraction) with 90% or 85% (v/v) ethanol as indicated. Each of the three extractions was performed by swirling the solids with a volume of ethanol solution equal to one-third of the original volume of culture filtrate. The three extracts prepared in this manner were combined, taken to dryness *in vacuo* as described above, dissolved in a volume of 76% (v/v) ethanol equal to one-twentieth of the original volume of culture filtrate (20× concentration), and stored at 4°C for later use.

### Analytical thin-layer chromatography (TLC) procedures

Analytical TLC separations were performed on Avicel® Microcrystalline Cellulose Uniplates (5 × 20 cm, 250 μm layer, glass-backed) and on Hard-Layer Silica Gel GHL Uniplates (5 × 20 cm, 250-μm layer, glass-backed, with an inorganic binder). For chromatography on cellulose plates, the solvent consisted of ethyl acetate:isopropanol:water (7.5:15:10). For chromatography on silica GHL plates, the solvent consisted of ethyl acetate:isopropanol:methanol:water (5:5:18:2). Unless otherwise indicated, the chromatographic samples (200 μL of the test solution) were applied to an origin line located 3 cm from one end of the plate as previously described [11]. The chromatograms were developed over a distance of 12 cm from the origin. The developed chromatograms were dried and sprayed with a ninhydrin solution consisting of 0.25% (w/v) ninhydrin dissolved in 95% (v/v) ethanol containing 3.0 mL of glacial acetic acid per 100 mL of final solution. Color development was achieved by heating the sprayed chromatograms in an oven at 80-90°C for 15 min.

The distribution of antimicrobial activity on the cellulose TLC chromatograms was determined in our standard agar diffusion assay. For this purpose, the chromatogram was divided into twelve 1-cm zones located between the origin and the solvent front. The cellulose from each zone (1 × 5 cm area) was scraped into separate 2.0-mL microfuge tubes, suspended in 1.33 mL of deionized water, and vortexed repeatedly to give a solution representing a 3-fold concentration relative to the original culture filtrate. The cellulose was pelleted by centrifugation (10,000 rpm, 10 min, Sorvall MC 12V Minifuge), and the supernatant from each tube was filter sterilized (0. 2 μm Acrodisc 13 mm syringe filter) prior to testing in the agar diffusion assay.

### Sephadex G-15 column chromatography

Sephadex G-15 (107 grams, medium grade) was swollen in deionized water and packed into a column (2.5 × 68 cm, 335 mL bed volume) in the same solvent. The column was washed extensively with deionized water prior to initial sample loading and between column runs. Details of column fractionations are given in the legends to the corresponding figures.

### Chrome Azurol S assays of Sephadex G-15 column fractions

Aliquots of Sephadex G-15 column fractions were assayed for phosphate (a major contaminant from the medium) and for amino acids using Fe-CAS and Cu-CAS reagents respectively. (The specificities of these reagents are illustrated in Additional files 5 and 6.) The reagents, prepared according to Shenker *et al*. [44]*,* were composed of 210 μM CAS and 200 μM of either CuSO_4_ or FeSO_4_ in 40 mM MES buffer. The resulting solutions were adjusted to either pH 5.5 (Cu-CAS) or 5.7 (Fe-CAS) with NaOH. To assay column fractions for metal binding, 75 μL of the appropriate CAS reagent was mixed with 75 μL of a column fraction in each well of a 96-well microtiter plate (Corning Costar No. 3590). The resulting absorbance was measured at 595 nm in a Bio-Tek EL808 plate reader. The presence of compounds that competed with CAS for metal binding caused a reduction in absorbance. Changes (reductions) in absorbance were measured relative to the most strongly absorbing fraction in the column profile and plotted as indicated.

### Preparative TLC procedures

Preparative TLC separations were performed on Avicel® Microcrystalline Cellulose Plates (20 × 20 cm, 1000 μm layer). Prior to use, preparative plates were washed by ascending chromatography in deionized water (twice) followed by one wash with redistilled 95% ethanol. The plates were dried overnight between washings. The chromatographic samples consisted of 2.0-mL aliquots per plate of a concentrated 76% ethanol solution (40× concentration) of the solids from the main Cu-binding peak of the Sephadex G-15 fractionation described in the text. The Analtech TLC Sample Streaker was used to apply the sample by repeated streaking across an origin line located 3 cm from the end of the plate. A filtered air-stream was used to dry the origin for 20 to 30 seconds between applications. Following the last application and prior to development, the plates were allowed to air dry for 8 minutes outside the stream. The plates were developed in 76% (v/v) ethanol (250 mL solvent in rectangular tanks, dimensions *ca.* 30L × 10W × 26 cm H) over a distance of 12 cm, dried, and examined under UV light (254 nm). Preliminary experiments determined that the ninhydrin-reactive compound of interest was localized in a narrow band (*ca.* 1 cm diameter, *ca.* R_f_ 0.55) delineated at its leading margin by a narrow UV-absorbing band and bounded at its trailing edge by a narrow fluorescent band immediately preceding a broader UV-absorbing band. These bands were used as markers in purifying the compound by preparative chromatography.

Eight preparative thin-layer plates were used to fractionate the 40× solution of the material recovered from Sephadex G15 column. The plates were developed with 76% ethanol. For each chromatogram, the area between the UV-absorbing and UV-fluorescent marker bands was scraped into separate 30-mL Corex centrifuge tubes. Deionized water (10 mL per tube) was added to each tube. After the tubes were vortexed (3 min), an additional 10 mL of deionized water was added to each tube, and the tubes were centrifuged at 6800 × g in a Sorvall SS34 rotor. The supernatants were decanted, pooled, filter sterilized [0. 2-μm, 25-mm Acrodisc syringe filter (Pall Life Sciences, Ann Arbor, MI)], and stored at 4°C prior to final purification by Sephadex G-15 column chromatography.

### Structural analysis of purified compound

NMR data were acquired on a Bruker DRX 300 MHz spectrometer equipped with a 5 mm BBO NMR probe. Chemical shifts were referenced to tetramethylsilane (δ_H_, δ_C_; 0, 0 ppm) in D_2_O. HRESIMS data were acquired in positive mode on an AB SCIEX Triple TOF 5600 spectrometer. Optical rotation was measured on a JASCO P-1010 polarimeter.

The observed and calculated HRESIMS and chemical shifts for L-(+)-Furanomycin [(2*S*,2′*R*,5′*S*)-2-Amino-2-(5′-methyl-2′,5′-dihydrofuran-2′-yl)acetic Acid, 1] are as follows: HRESIMS(+) obsd [M+H]^+^*m/z* 158.0812 (calcd for C_7_H_11_ON_3_, 158.0801); ^1^H NMR (300 MHz, D_2_O) 6.07 (1H, dt, *J* = 6.2, 1.8 Hz, H-4′), 5.74 (1H, dt, *J* = 6.2, 1.8 Hz, H-3′), 5.34 (1H, m), 5.00 (1H, p, *J* = 6.2 Hz, H-2′), 3.75 (1H, d, *J* = 2.5 Hz, H-2), 1.14 (1H, d, *J* = 6.4 Hz, H-6′); ^13^C NMR (75 MHz, D_2_O) 172.3 (C, C-1), 136.3 (CH, C-4′), 124.3 (CH, C-3′), 84.31 (CH, C-2′), 84.24 (CH, C-5′), 57.5 (CH, C-2), 21.0 (CH_3_, C-6′).

## Abbreviations

GAF: Germination Arrest Factor; FVG: 4-formylaminooxyvinylglycine; TLC: Thin-layer chromatography; NMR: Nuclear magnetic resonance; HRESIMS: High-resolution electrospray mass spectrometry; MVG: Methoxyvinylglycine; CAS: Chrome Azurol S; MES: 2-(N-morpholino)ethanesulfonic acid

## Competing interests

The authors declare that they have no competing interests.

## Authors’ contributions

KT carried out all of the microbiological testing and drafted the manuscript. KM carried out the NMR and other aspects of the structural analyses. MA prepared culture filtrates and carried out the germination assays. DA purified the sample of the compound used for structural analysis. GB participated in the design and coordination of the study and helped draft the manuscript. All of the authors have read and approved the manuscript.

## Supplementary Material

Additional file 1 **Examples of the observed effects of *P. fluorescens* SBW25 culture filtrate on the growth of lawns of selected bacterial strains.** Images of representative agar diffusion assays are shown for five strains of plant pathogens that were sensitive to the filtrate and one representative of strains that did not respond to the filtrate (lower right corner). Click here for file

Additional file 2 ^1^H NMR spectrum of the purified ninhydrin-reactive fraction containing L-furanomycin. Click here for file

Additional file 3 ^13^C NMR spectrum of the purified ninhydrin-reactive fraction containing L-furanomycin. Click here for file

Additional file 4 **Effects of selected amino acids on the antimicrobial activity of *P. fluorescens* SBW25 culture filtrate.** Images of representative agar diffusion assay plates are shown for assays in which the indicated amino acids were added to *P. fluorescens* SBW25 culture filtrate at a final concentration of 10 mM, and aliquots of the resulting solutions were then tested for antimicrobial activity against *Dickeya dadantii*. Click here for file

Additional file 5 **Specificity of the Chrome Azurol assay.** Quantitative data for the reactions of the Cu and Fe ChromeAzurol reagents with various known compounds are shown. Click here for file

Additional file 6 **Additional tests of the specificity of the Chrome Azurol assay.** Quantitative data for the reactions of the Cu and Fe ChromeAzurol reagents with various additional known compounds are shown. Click here for file
