# Supplementary material for: Pseudomonas fluorescens SBW25 produces furanomycin, a non-proteinogenic amino acid with selective antimicrobial properties
Source: BMC Microbiol. 2013 May 20;13:111. doi: 10.1186/1471-2180-13-111 (PMC3662646; doi:10.1186/1471-2180-13-111)
Supplement: Additional file 1 — Examples of the observed effects of P. fluorescens SBW25 culture filtrate on the growth of lawns of selected bacterial strains. Images of representative agar diffusion assays are shown for five strains of plant pathogens that were sensitive to the filtrate and one representative of strains that did not respond to the filtrate (lower right corner). [file 1471-2180-13-111-S1.pdf]

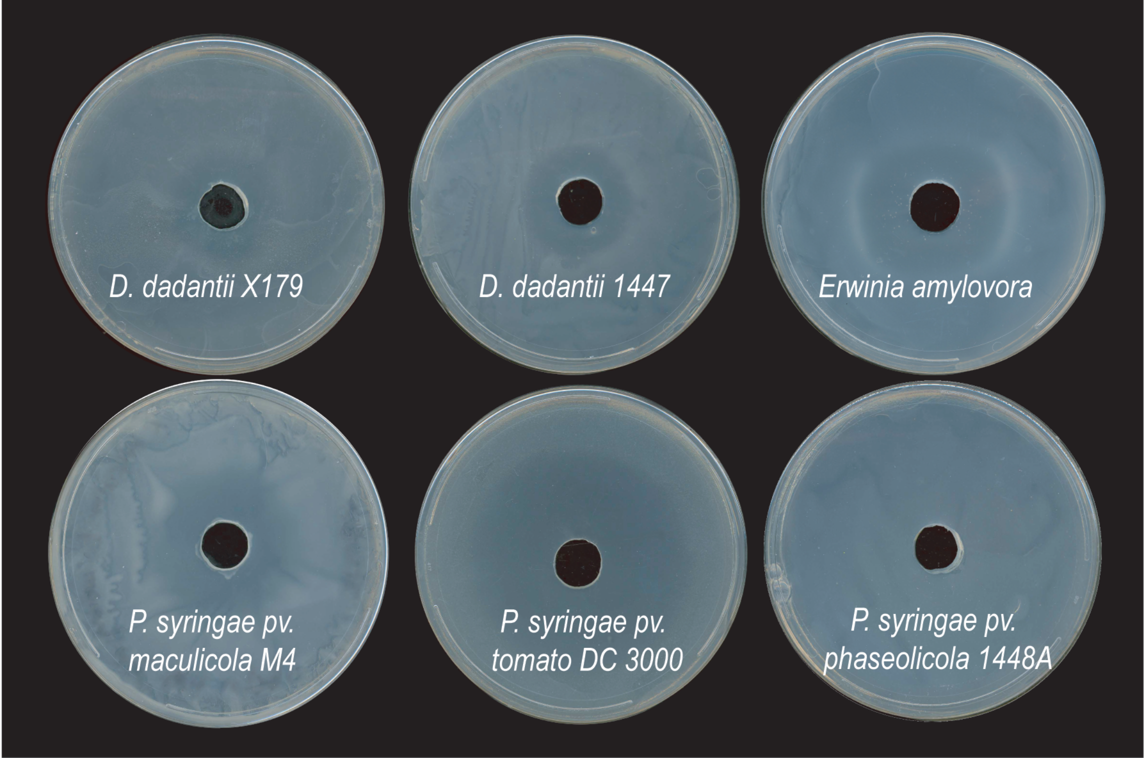
D. dadantii X179
D. dadantii 1447
Erwinia amylovora
P. syringae pv. maculicola M4
P. syringae pv. tomato DC 3000
P. syringae pv. phaseolicola 1448A
