# Supplementary figures and images for: Pseudomonas fluorescens SBW25 produces furanomycin, a non-proteinogenic amino acid with selective antimicrobial properties
Source: BMC Microbiol. 2013 May 20;13:111. doi: 10.1186/1471-2180-13-111 (PMC3662646; doi:10.1186/1471-2180-13-111)

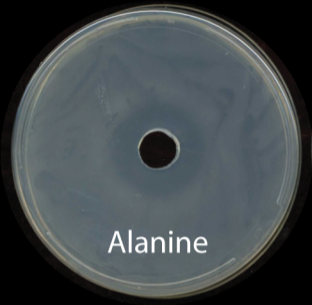

Alanine

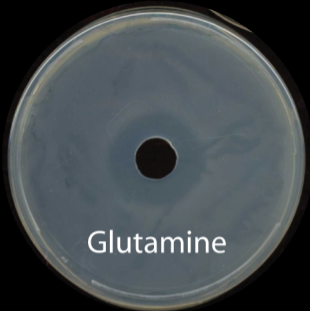

Glutamine

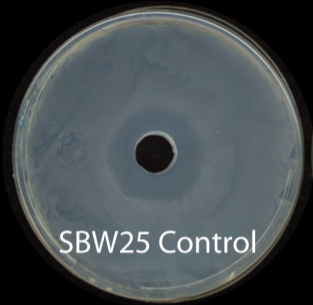

SBW25 Control

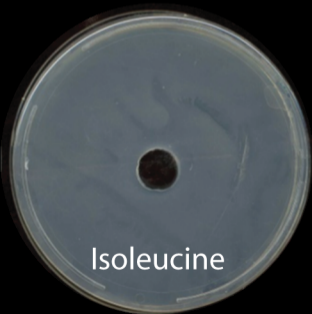

Isoleucine

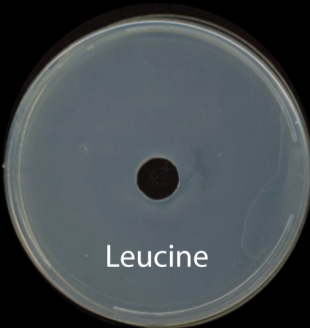

Leucine

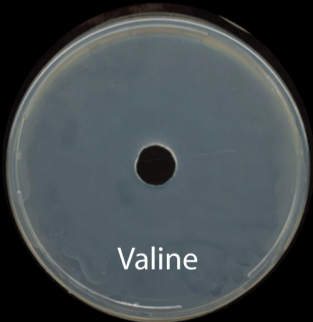

Valine

Supplement: Additional file 2 — 1H NMR spectrum of the purified ninhydrin-reactive fraction containing L-furanomycin. [file 1471-2180-13-111-S2.pdf]
