# Supplementary material for: Pseudomonas fluorescens SBW25 produces furanomycin, a non-proteinogenic amino acid with selective antimicrobial properties
Source: BMC Microbiol. 2013 May 20;13:111. doi: 10.1186/1471-2180-13-111 (PMC3662646; doi:10.1186/1471-2180-13-111)
Supplement: Additional file 3 — 13C NMR spectrum of the purified ninhydrin-reactive fraction containing L-furanomycin. [file 1471-2180-13-111-S3.pdf]

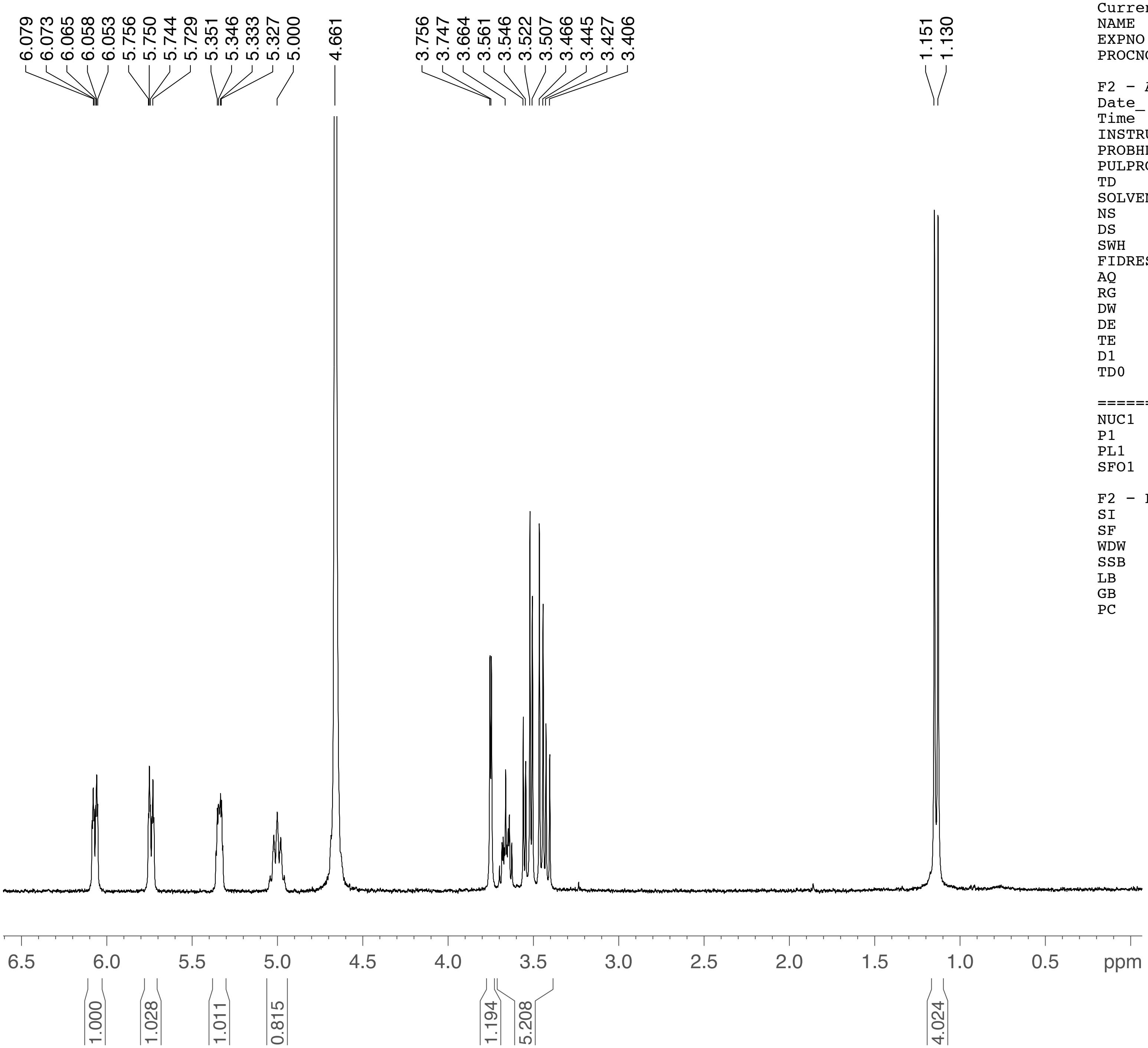


```
Current Data Parameters
NAME       DAXV47_SBW25
EXPNO                 1
PROCNO                1

F2 - Acquisition Parameters
Date_          20110924
Time              11.26
INSTRUM          DRX300
PROBHD   5 mm BBO BB-1H
PULPROG            zg30
TD                65536
SOLVENT             D2O
NS                   32
DS                    4
SWH            5387.931 Hz
FIDRES         0.082213 Hz
AQ            6.0817909 sec
RG                812.7
DW               92.800 usec
DE               158.64 usec
TE                298.1 K
D1           1.60000002 sec
TD0                   1

======== CHANNEL f1 ========
NUC1                 1H
P1                11.45 usec
PL1                0.00 dB
SFO1        300.1319508 MHz

F2 - Processing parameters
SI                32768
SF          300.1300105 MHz
WDW                  EM
SSB                   0
LB                 0.30 Hz
GB                    0
PC                 1.00
```
