# Supplementary material for: Pseudomonas fluorescens SBW25 produces furanomycin, a non-proteinogenic amino acid with selective antimicrobial properties
Source: BMC Microbiol. 2013 May 20;13:111. doi: 10.1186/1471-2180-13-111 (PMC3662646; doi:10.1186/1471-2180-13-111)
Supplement: Additional file 4 — Effects of selected amino acids on the antimicrobial activity of P. fluorescens SBW25 culture filtrate. Images of representative agar diffusion assay plates are shown for assays in which the indicated amino acids were added to P. fluorescens SBW25 culture filtrate at a final concentration of 10 mM, and aliquots of the resulting solutions were then tested for antimicrobial activity against Dickeya dadantii. [file 1471-2180-13-111-S4.pdf]

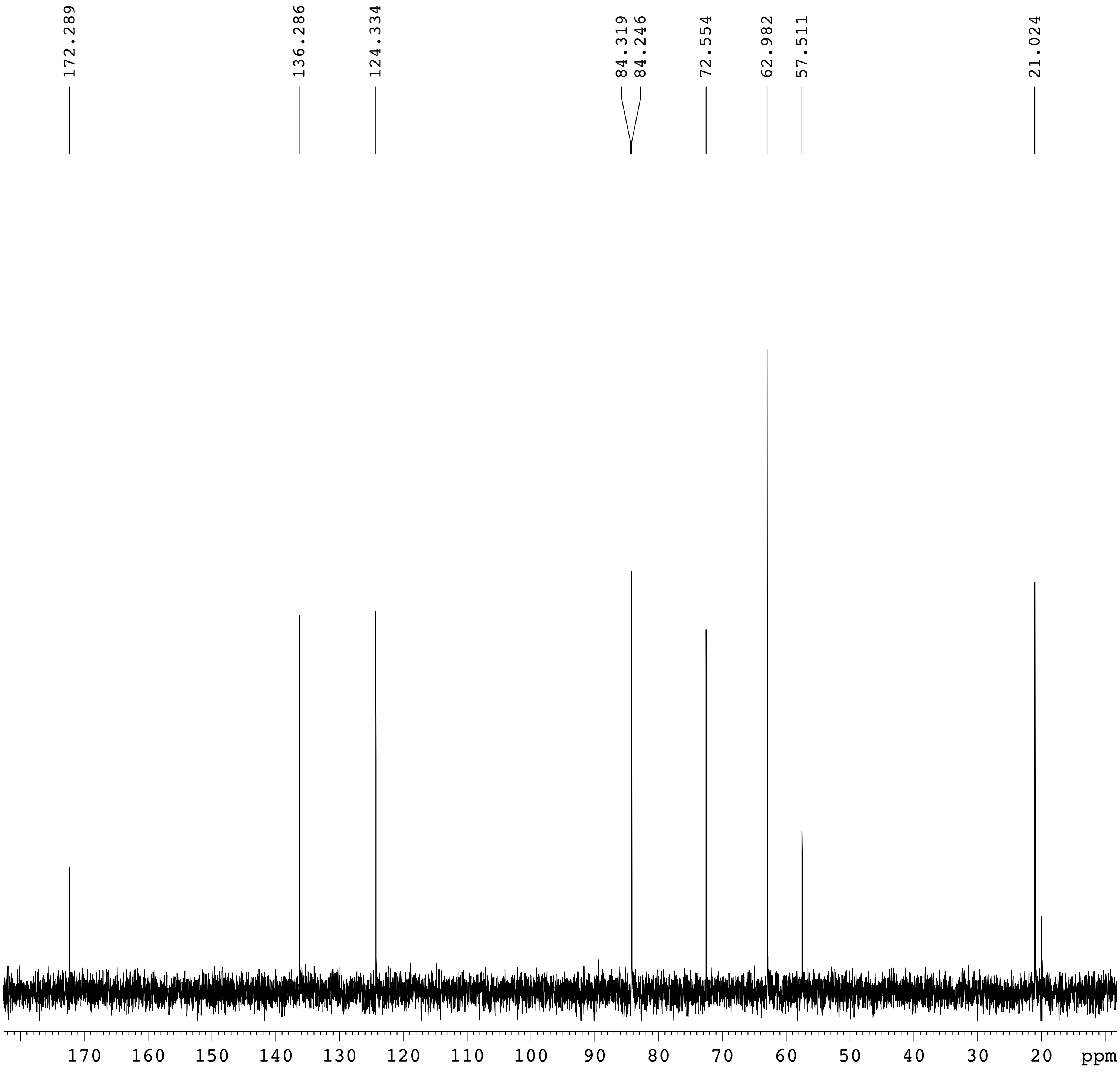

Current Data Parameters
NAME DAXV47_SBW25
EXPNO 101
PROCNO 1

F2 - Acquisition Parameters
Date_ 20110924
Time 12.18
INSTRUM DRX300
PROBHD 5 mm BBO BB-1H
PULPROG zgpg30
TD 65536
SOLVENT CDCl3
NS 6810
DS 8
SWH 22727.273 Hz
FIDRES 0.346791 Hz
AQ 1.4418420 sec
RG 8192
DW 22.000 usec
DE 6.00 usec
TE 298.3 K
D1 0.30000001 sec
d11 0.03000000 sec
DELTA 0.20000002 sec
TD0 1

======== CHANNEL f1 ========
NUC1 13C
P1 8.95 usec
PL1 0.00 dB
SFO1 75.4783145 MHz

======== CHANNEL f2 ========
CPDPRG2 waltz16
NUC2 1H
PCPD2 85.00 usec
PL2 0.00 dB
PL12 19.00 dB
PL13 19.00 dB
SFO2 300.1312005 MHz

F2 - Processing parameters
SI 32768
SF 75.4677106 MHz
WDW EM
SSB 0
LB 1.00 Hz
GB 0
PC 3.00
