# Supplementary material for: Pseudomonas fluorescens SBW25 produces furanomycin, a non-proteinogenic amino acid with selective antimicrobial properties
Source: BMC Microbiol. 2013 May 20;13:111. doi: 10.1186/1471-2180-13-111 (PMC3662646; doi:10.1186/1471-2180-13-111)
Supplement: Additional file 5 — Specificity of the Chrome Azurol assay. Quantitative data for the reactions of the Cu and Fe ChromeAzurol reagents with various known compounds are shown. [file 1471-2180-13-111-S5.pdf]

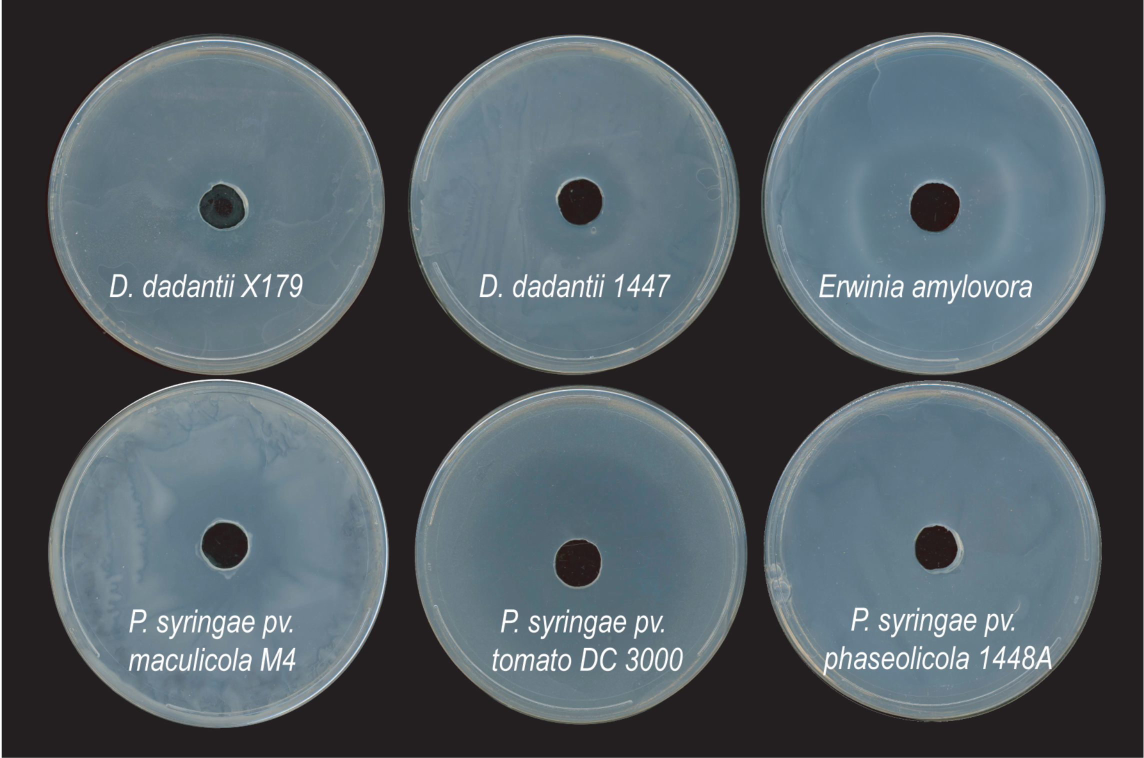
D. dadantii X179
D. dadantii 1447
Erwinia amylovora
P. syringae pv. maculicola M4
P. syringae pv. tomato DC 3000
P. syringae pv. phaseolicola 1448A
